# Estimating How Inflated or Obscured Effects of Climate Affect Forecasted Species Distribution

**DOI:** 10.1371/journal.pone.0053646

**Published:** 2013-01-11

**Authors:** Raimundo Real, David Romero, Jesús Olivero, Alba Estrada, Ana L. Márquez

**Affiliations:** 1 Biogeography, Diversity, and Conservation Research Team, Department of Animal Biology, Faculty of Sciences, University of Malaga, Malaga, Spain; 2 Instituto de Investigación en Recursos Cinegéticos IREC, (CSIC-UCLM), Ciudad Real, Spain; University of Western Australia, Australia

## Abstract

Climate is one of the main drivers of species distribution. However, as different environmental factors tend to co-vary, the effect of climate cannot be taken at face value, as it may be either inflated or obscured by other correlated factors. We used the favourability models of four species (*Alytes dickhilleni, Vipera latasti, Aquila fasciata* and *Capra pyrenaica*) inhabiting Spanish mountains as case studies to evaluate the relative contribution of climate in their forecasted favourability by using variation partitioning and weighting the effect of climate in relation to non-climatic factors. By calculating the pure effect of the climatic factor, the pure effects of non-climatic factors, the shared climatic effect and the proportion of the pure effect of the climatic factor in relation to its apparent effect (ρ), we assessed the apparent effect and the pure independent effect of climate. We then projected both types of effects when modelling the future favourability for each species and combination of AOGCM-SRES (two Atmosphere-Ocean General Circulation Models: CGCM2 and ECHAM4, and two Special Reports on Emission Scenarios (SRES): A2 and B2). The results show that the apparent effect of climate can be either inflated (overrated) or obscured (underrated) by other correlated factors. These differences were species-specific; the sum of favourable areas forecasted according to the pure climatic effect differed from that forecasted according to the apparent climatic effect by about 61% on average for one of the species analyzed, and by about 20% on average for each of the other species. The pure effect of future climate on species distributions can only be estimated by combining climate with other factors. Transferring the pure climatic effect and the apparent climatic effect to the future delimits the maximum and minimum favourable areas forecasted for each species in each climate change scenario.

## Introduction

Species distribution models (SDMs) are becoming increasingly important tools for conservation biology, because determining which factors drive the distribution patterns can help to adopt more specific and appropriate strategies for the management and conservation species [1]. This knowledge is also the basis for making good forecasts on the effect of climate change on future species distributions or suitable areas, which is a new challenge for environmental managers [2, 3]. However, the estimation of impact of the climate change on future species distribution is complex and related to different kinds of uncertainties [4–9], including the inability to assess the weight of climate as a driver of species distribution.

Climate envelope models are widely used to forecast future species distributions under climate change scenarios [10–13]. Some authors argue against the validity of using SDMs based on climatic variables alone as tools to forecast future species distributions, because they consider that other factors play a role in the distributions and that these factors are not taken into account in the models [3, 5, 14–16]. Apart from climate, species distributions may be controlled by spatial trends, topography, human activity, biotic interactions, history, and population dynamics, among others [17–20]. As species may show differential responses to these factors [3, 5, 21], their combined importance should be assessed before projecting species distribution models to the future. In addition, the effect of climate can only be assessed in the context of the other influential factors, because its pure effect on species distributions could be obscured or overrated by correlated aspects, becoming evident only when all the relevant factors are considered together [18, 19, 22, 23].

Variation partitioning techniques have been used to separate the effects of different factors on species richness [24, 25], on abundance [26], on ecological communities [27], on species assemblages [28] and also on species distributions [29, 30]. These techniques have also been used to segregate the pure effect of different factors (topography, climate, human activity, spatial situation and lithology) on species distributions [31]. However, these techniques have not been used to relate the pure climatic effect to its apparent effect, being the latter in correlation with other factors. This is of fundamental importance, because the apparent climatic effect could be misrepresenting the true role of climate on species distributions due to the effect of other correlated factors. Therefore, the potential changes in species distributions related to climate change could be distorted and lead to misleading conclusions about the species vulnerability or their risk of extinction.

Mountains are areas of interest regarding the early detection and study of the signals of climate change and its impact on ecological systems [32]. Mountain species are particularly sensitive to climate change [33–36], because mountain areas have more pristine habitats than lowland landscapes, and because these species can track climate change over shorter distances [37, 38]. Predicting the possible effects of climate change on the future distributions of these kinds of species is of fundamental importance in conservation plans.

We evaluated the relative contribution of climate in mainland Spain to the forecasted favourability of four vertebrate mountain species whose distributions are related to climate and to altitude or slope, and for which published distribution models are available both for the present and for the future according to the apparent effect of climate [8, 19]. The aim of this work is to propose a method by which to analyze the relative contribution of climate in relation to non-climatic factors (spatial, topographic, and human) and to distinguish between its apparent and its pure effect in models designed to forecast how favourable areas for species could vary because of climate change. Our results underline the possibly misleading outcome of not considering the pure climatic effect in the projections of the SDMs to the future.

## Methods

### The study area

Mainland Spain is located in southwestern Europe and covers an area of 493,518 km^2^. Its latitude (40° N), geographical position and complex orography make its climate heterogeneous, with a precipitation gradient (100–2500 mm) decreasing mainly eastward and southward, and a temperature gradient (6°–18°C) decreasing mainly northward [39]. In Spain, five homogeneous climatic precipitation regions [39] can be distinguished: 1) the North Atlantic coast, which has abundant and regular precipitation due to the continuous arrival of Atlantic frontal systems; 2) the central area that receives wet and cold air intrusions from frontal Atlantic systems and presents low precipitations; 3) the eastern coast, which is characterized by irregular and scanty annual precipitation, with large variability due to severe rainfall events produced by wet and warm air intrusions from the Mediterranean Sea [40]; 4) the southeastern region, which is a dry desert-like area with very little rainfall; and 5) the southwestern region, which has more regular and abundant rainfall influenced by Atlantic winds.

This makes Spain particularly appropriate for analyzing the effect of different climate change scenarios on species distributions (e.g., [19]).

### The species

We analysed the distribution in mainland Spain of four vertebrate species whose distributions are positively associated with altitude or slope. We chose an amphibian (Baetic midwife toad, *Alytes dickhilleni*), a reptile (Lataste’s viper, *Vipera latasti*), a bird (Bonelli’s eagle, *Aquila fasciata*) and a mammal (Iberian wild goat, *Capra pyrenaica*). *A. dickhilleni* is a small toad, between 32.8 and 56.5 mm in length, endemic to Spain and located exclusively in the mountainous systems of the southwestern part of the Iberian Peninsula. It lives on rough and steep terrains, in cracks and crevices next to streams, springs and pools. Species reproduction occurs in permanent water points. *V. latasti* is a venomous viper species found in southwestern Europe and northwestern Africa, which can reach 70 cm in length. Its distribution in Spain is relegated by human activity to mountainous and sparsely populated areas. *A. fasciata* is a small to medium-size eagle, 55–65 cm in length. It is one of the rarest raptors in Europe and is a priority target species for special conservation measures in Spain (Council Directive 2009/147/EC and National Real Decreto 439/1990). It occupies mountain ranges, small hills and plains, where it breeds mainly in cliffs. *C. pyrenaica* is an endemic goat, only found in the mountainous areas of Spain. It is a species with strong sexual dimorphism, males are larger than females and their horns are three times longer and thicker than those of females. It lives in both forests and grassy expanses in mountains at altitudes between 500 and 2500 meters.

### Baseline models

As starting point, we used the current favourability models obtained for *A*. *dickhilleni, V. latasti, A. fasciata* and *C. pyrenaica* using different climate change scenarios in mainland Spain, available in Márquez *et al.* [19]. The distributions of the four species were modelled using variables related to climate, spatial situation, topography, and human activity (see Table S1 for more specific details of the variables) and with the favourability function as the modelling technique [41–43]. Climatic variables were provided by the Agencia Estatal de Meteorología (AEMET), which regionalized the general circulation models CGCM2 (Canadian Climate Centre for Modeling) and ECHAM4 (Max Planck Institut für Meteorologie) and the A2 and B2 emission scenarios for Spain. Mean values of the climatic variables were obtained for the periods: 1961–1990, 2011–2040, 2041–2070 and 2071–2100 (Figure S1). For each species a total of four factor models were obtained, related to climate alone, spatial situation alone, topography alone, and human activity alone, respectively. A combined model was then obtained which took into account the four environmental factors (climatic and non-climatic) together (see Márquez *et al.* [19] for more details). In this way, the favourability values for each species in each cell at the present time (*F_p_*) were obtained. Future favourability values for each species according to the apparent effect of climate (*F_fClim_*) in each cell, as well as an analysis of the impact of climate model choice and scenario choice on expected favourability, were obtained by Real *et al.* [8] by replacing the current (1961–1990) climatic values in the combined favourability models with those expected according to each AOGCM and SRES for the following time periods (2011–2040, 2041–2070, 2071–2100).

### Variation partitioning

We segregated the pure effect of climate from the effect of the other factors in the models using a variation partitioning procedure similar to that of Borcard *et al.* [44], Barbosa *et al.* [29] and Muñoz *et al.* [45] with some modifications. Thus, we specified how much of the variation of the combined favourability model was explained by the pure effect of climate (not affected by the covariation with other factors in the model), and which proportion of the climate effect cannot be distinguished from that of the other factors (shared effect) [45].

The portion of the variation in the model apparently explained by climate was estimated using the coefficient of determination of the linear regression of the logit function (*y*) of the model on the climatic variables included in it (R^2^_Clim_) for the period 1961–1990; the part apparently explained by the non-climatic factors (R^2^_NClim_) was obtained in a similar manner. The pure effect of climate (R^2^_pClim_) was then assessed by subtracting from 1 the variation of the combined model explained by the non climatic factors (R^2^_pClim_  =  1 - R^2^_NClim_). The pure effect of the non-climatic factors was obtained by subtracting from 1 the variation explained by climate (R^2^_pNClim_  =  1 - R^2^_Clim_). The effect shared by climate and non-climatic factors was obtained by subtracting from 1 both pure effects (R^2^_ClimNClim_  =  1 - R^2^_pClim_ - R^2^_pNClim_). We used the adjusted R^2^ in all cases [46] although, given the high number of cells used (n = 5167 10x10 km^2^ UTM cells), the difference between R^2^ and adjusted R^2^ was very small. This partitioning procedure was applied only to the portion of variation explained by the model, not over the total variation of the species distribution [44, 46], as it is the explanatory model which is calibrated to be transferred to the future. We expressed these effects as percentages and considered them to be the percentage of model variation attributable to the pure climatic effect (PCE), the pure non-climatic effect (PNCE) and the shared effect of climate and non-climatic factors (SCE).

We estimated the proportion of the apparent climatic effect represented by the pure effect of climate as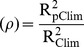. We calculated the logit function expected for the future in each cell according to the pure effect of climate (*y*_fClim_) by applying the expression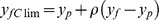, where *y_p_* is the logit function at the present time and *y*_f_ is the logit function expected for the future according to the apparent effect of climate. We obtained the future favourability according to the pure climatic effect (*F_fPClim_*) using the expression:



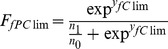



where *n*_1_ is the number of presences and *n*_0_ the number of absences (see formula 7 in Real *et al.* [41]).

This way of inferring the effect of climate differs from usual projections according to climate change scenarios, which are customarily based on the apparent effect of climate. The difference between the sum of areas forecasted to be favourable according to the apparent climatic effect (F_AC_), calculated using the expression
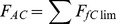
, and those forecasted according to the pure climatic effect (F_PC_), calculated using the expression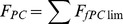, was computed and expressed as the relative proportion of discrepancy R = (*F_AC_*-*F_PC_* )/*F_AC_*.

## Results

Climate had a more significant effect than non-climatic factors on *A. fasciata* (Table 1). However, for the other species (*A. dickhilleni, V. latasti and C. pyrenaica*), the non-climatic effect was more important than the climatic effect (Table 1). In addition, regarding *A. dickhilleni,* the percentage of variation of the model attributable to a Shared Climatic Effect (SCE) was very important, which means that the apparent effect of climate could be due in large part to other correlated factors. Regarding *A. fasciata* and *C. pyrenaica,* the values of SCE were negative in the majority of the models. These negative values measure the amount of reciprocal obscuring caused by factors that have opposite geographic effects on the explained favourability, so that in these species the apparent climatic effect under-represents the pure climatic effect.

**Table 1:** Variation partitioning of the combined favourability models for the period 1961–1990.

AOGCM-SRES		*A. dickhilleni*	*V. latasti*	*A. fasciata*	*C. pyrenaica*
CGCM2-A2	PNCE	36.20	56.13	67.07	78.63
	PCE	6.80	24.19	66.73	38.40
	SCE	57.00	19.68	−33.80	−17.03
	ρ	0.107	0.551	2.026	1.797
CGCM2-B2	PNCE	37.27	59.27	57.78	77.99
	PCE	7.02	37.62	72.82	37.82
	SCE	55.71	3.10	−30.60	−15.80
	ρ	0.112	0.924	1.725	1.718
ECHAM4-A2/B2	PNCE	66.43	43.61	46.92	80.08
	PCE	12.11	19.45	79.28	6.40
	SCE	21.46	36.94	−26.19	13.53
	ρ	0.361	0.345	1.493	0.321

Values shown are the percentages of variation explained by the Pure Non-Climatic Effect (PNCE), the Pure Climatic Effect (PCE) and the Shared Climatic Effect (SCE). ρ: Proportion of pure climatic factor in relation to the whole climatic factor.

**Figure 1:**
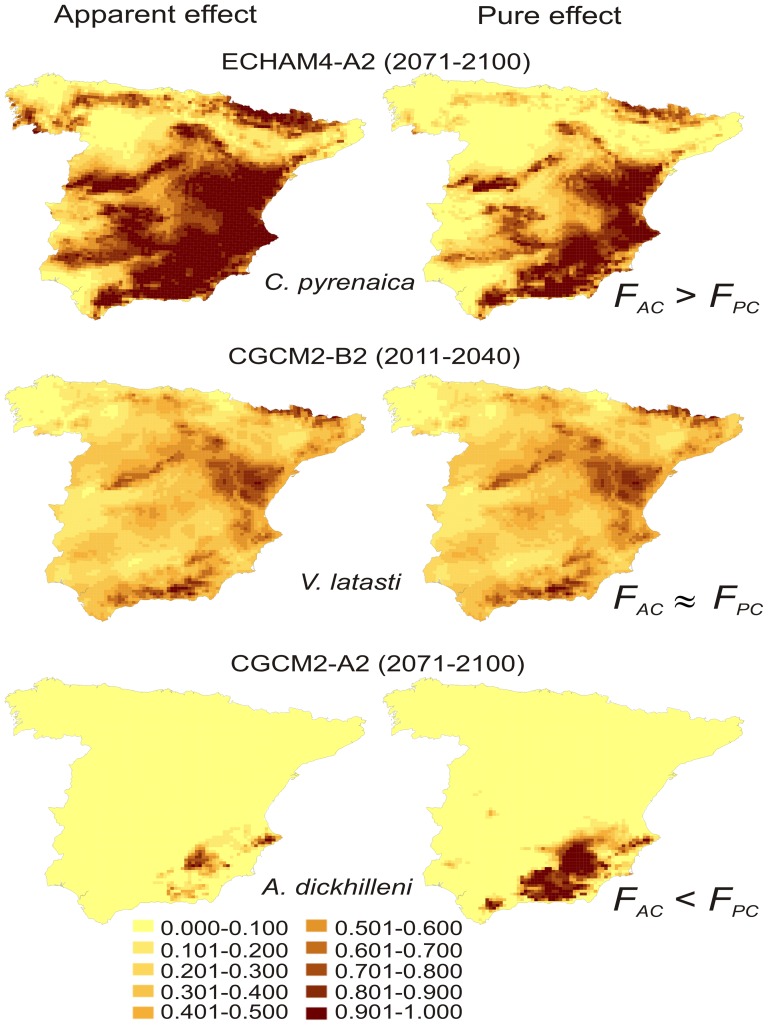
Forecasted favourability. Distribution of the future favourability forecasted according to the apparent climatic effect (F_AC_) and that forecasted according to the pure climatic effect (F_PC_). These maps represent three situations: F_AC_ < F_PC_, F_AC_ 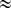 F_PC_, F_AC_ > F_PC._ The examples shown are those where the difference or similarity were most evident.

**Figure 2:**
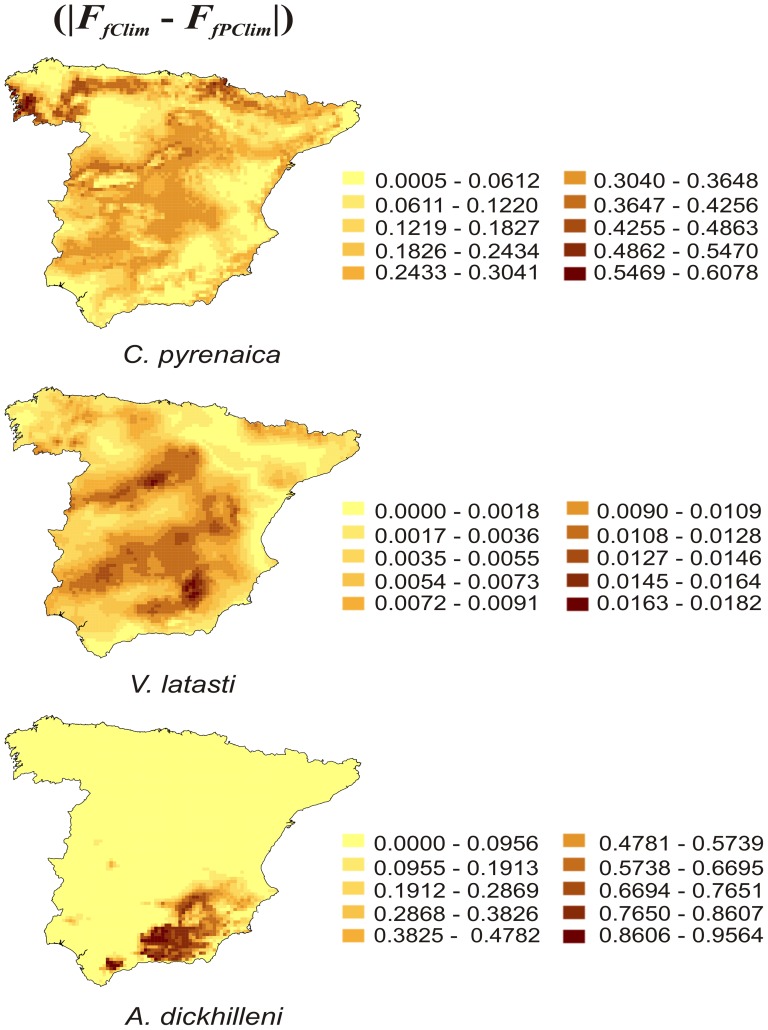
Differences between forecasted local favourabilities. Distribution of the uncertainty associated with differences between the favourabilities forecasted according to the apparent and the pure climatic effect (|*F_fClim_* - *F_fPClim_*|) for the three species and situations represented in Figure 1.

Figure 1 shows three examples of the differences between forecasted favourabilities taking into account the apparent climatic effect (*F_AC_*) and the pure climate effect (*F_PC_*), when *F_PC_* is lower, similar or higher than *F_AC_*. The differences between the two future forecasted favourabilities (*F_AC_* - *F_PC_*) and the relative proportion of discrepancy between both types of effects (R =  (*F_AC_* - *F_PC_*) / *F_AC_*) are shown in Table 2. Figure 2 shows the spatial distribution of the difference between the forecasted favourabilities taking into account the apparent climatic effect and the pure climatic effect (|*F_fClim_* - *F_fPClim_*|) for the species and situations described in Figure 1.

**Table 2:** Differences between the favourability forecasted according to the apparent climatic effect (*F_AC_*) and to the pure climatic effect (*F_PC_*) for each species and period of time.

		*A. dickhilleni*		*V. latasti*		*A. fasciata*		*C. pyrenaica*	
	AOGCM-SRES	*F_AC_*-*F_PC_*	R	*F_AC_*-*F_PC_*	R	*F_AC_*-*F_PC_*	R	*F_AC_*-*F_PC_*	R
**2011–2040**	**CGCM2-A2**	21.874	0.043	−501.387	−0.518	−339.467	−0.151	−429.507	−0.209
	**CGCM2-B2**	68.640	0.122	−30.569	−0.017	−353.400	−0.146	−413.958	−0.204
	**ECHAM4-A2**	241.694	0.297	−385.007	−0.255	−215.186	−0.092	192.548	0.092
	**ECHAM4-B2**	282.001	0.303	−279.382	−0.167	−336.028	−0.125	216.448	0.121
**2041–2070**	**CGCM2-A2**	−150.240	−0.489	−416.164	−0.339	−721.372	−0.277	−753.816	−0.294
	**CGCM2-B2**	−28.377	−0.063	−62.350	−0.048	−517.740	−0.195	−531.307	−0.230
	**ECHAM4-A2**	216.533	0.270	−406.433	−0.277	−656.160	−0.158	406.952	0.197
	**ECHAM4-B2**	15.849	0.034	50.292	0.023	−724.439	−0.202	559.879	0.246
**2071–2100**	**CGCM2-A2**	−335.978	−4.230	−328.051	−0.225	−864.559	−0.309	724.801	0.176
	**CGCM2-B2**	−269.066	−1.262	512.495	0.173	−613.793	−0.217	−551.656	−0.237
	**ECHAM4-A2**	−9.056	−0.022	350.620	0.133	−285.609	−0.114	943.755	0.335
	**ECHAM4-B2**	148.574	0.227	214.544	0.089	−578.274	−0.177	813.794	0.311
**Mean absolute percentage of change**			61.33		18.86		18.05		22.10

R: Relative proportion of change ((*F_AC_* - *F_PC_*) / *F_AC_*).

## Discussion

The inclusion of climatic and non-climatic factors in SDMs is recommended not only because it can improve fit and increase their predictive accuracy [47, 48], but also because the effect of climate can only be assessed in the context of the other influential factors [18, 19, 22, 23]. Our results show that the correlation of influential non-climatic factors with temperature and precipitation could either inflate or obscure the apparent effect of climate, and that this modification of the apparent effect of climate would remain hidden if non-climatic variables were not included in the SDM. Even the use of the latitude and longitude of every cell alone may pinpoint certain areas of origin, dispersion, or past vicariance events driving current distributions, which results in a historically-caused spatial pattern that may coincide with specific climatic characteristics [49]. Consequently, the true effect of climate should be assessed in the context of spatial influences both on species distributions and on climate [50]. We used human, topographic and spatial variables as non-climatic predictors that, although correlated with climatic variables, can influence species distributions for reasons not directly linked to climate [18]. It was by taking into account these non-climatic factors and removing their effects statistically that we identified the underlying pure climate-distribution relationships, which could then be used in forecasting their distribution shifts under climate change [3].

However, the inclusion of climatic variables together with non-climatic, static variables entails other kinds of problems. Stanton *et al.* [51] considered that static variables such as elevation, latitude or longitude may hinder the accuracy of future predictions, as the relationships between them and climatic variables is likely to change in the future, and that including such variables in the SDM is likely to result in models which underestimate the effects of climate change. Our results confirm that this may be the case, although these effects may be under- or over-estimated. Our procedure is a way to gauge these relationships and assess the maximum extent to which the current correlation between these static variables and climate may affect the climatic parameters in the SDM.

On the other hand, the shared climatic effect is equally attributable to climate or to other correlated factors, so in our current state of knowledge the exact effect of climate cannot be determined with precision, although it lies somewhere between the apparent effect and the pure effect. The uncertainty related to the differences between both effects vary spatially and their intensity depends on the species (see Figure 2). This kind of uncertainty is added to other sources of uncertainty associated with forecasting future species distributions [8, 52, 53], among them those derived from assuming that the species' climate tolerances will remain constant through time, which is one serious limitation to the customary use of SDMs. However, despite these uncertainties, the estimation of species range shifts is the basis to predict where the species are likely to move under different future conditions [54]. More reliable predictions of species distribution responses to future climate conditions depend on developing more rigorous statistical analyses of the available data and on the combination of different factors, as well as on placing limits on the different uncertainties involved in the scientific forecasting of future events [19]. Transferring the pure climatic effect and the apparent climatic effect to the future allows us to delimit the maximum and minimum effect of climate on the species distributions.

Most of the favourability models of all the species considered in this study included three or four factors (spatial situation, topography, human activity and climate) (see Table 3 in Márquez *et al.* [19]) that we summarized into a climatic factor and a non-climatic factor. Pure climatic effect (PCE), pure non-climatic effect (PNCE) and shared climatic effect (SCE) on the future favourable areas for *A. dickhilleni, V. latasti, A. fasciata* and *C. pyrenaica* differed substantially (see Table 1). For *A. dickhilleni, V. latasti* and *C. pyrenaica* the PNCE was more important than the PCE, which suggests that their future distributions will be more related to non-climatic environmental variables, such as biotic interactions, past human activities or past contingent events, than with the climatic factor [31, 55]. For *A. fasciata,* and in most scenarios for *C. pyrenaica,* the SCE was negative, that is, the climatic effect and the non-climatic effect can be reciprocally obscured by their opposite effect on the explained favourability (Table 1) [24, 46]. In these cases the apparent climatic effect under-represents the pure climatic effect. Future favourabilitiy for these cases taking into account the pure climatic effect would represent their maximum future favourable area, which is higher than that forecasted according to the apparent climatic effect (Table 2). The future areas favourable to *A. dickhilleni* forecasted according to the apparent climatic effect differed from those forecasted according to the pure climatic effect by 61% on average, which was the highest difference in the four species considered (Table 2). In this case, the apparent climatic effect was highly inflated by non-climatic factors.

The SCE could be a measure of uncertainty related to the complex interactions existing between climate and non-climatic factors. In some cases it represents the obscured climatic effect (when it has a negative value) and in other cases the inflated climatic effect (when it has a positive value). In any case, the SCE represents the uncertainty associated with the possibility of misunderstanding the effect of climate due to the effect of other correlated factors. This improves the usefulness of this kind of model for understanding species’ potential responses to climate change, although possible changes in species-environment correlations through time can, nevertheless, place a limit on the predictive performance of these models [56]. According to Pearson and Dawson [10], understanding the complex interaction between the many factors affecting distributions is needed for the performance of more realistic simulations of the effect of climate change on species distributions. Models that take the effect of climate at face value yield future potential favourable areas that are, depending on the species, overestimated or underestimated. Using the method proposed in this paper, models may more realistically assess the levels of potential threat or opportunities to species of climate change.

## Conclusion

In contrast to the tendency of not using correlated variables in spatial distribution models due to the possibility of the resulting coefficients can being unstable, we have to deal with the fact that in nature most factors are correlated; thus analyses that separate the pure and combined effect of the relevant factors should be performed. Given that the apparent effect of climate can be either inflated or obscured by other correlated factors, transferring both the pure climatic effect and the apparent climatic effect to the future allows us to delimit the maximum and minimum favourable areas forecasted for each species in each climate change scenario, thus permitting us to assess the uncertainty associated with the possibility of misrepresenting the effect of climate. This also allows us to detect and control the over- or under-estimation of the effect of climate change (either positive or negative) on future species distributions that is implicit in current climate envelope models. This may make models more complex and harder to perform, but the output would be closer to what may be scientifically forecasted taking into account this kind of uncertainty.

## Supporting Information

Figure S1 **Precipitations and temperatures.** Annual precipitations and mean annual maximum temperatures for each period and combination of circulation model and scenario used.
(TIF)

Table S1 Factors and variables. Explanatory factors and variables used to model the species distributions of Márquez et al 2011 [19].
(DOC)
