# Supplementary figures and images for: Estimating How Inflated or Obscured Effects of Climate Affect Forecasted Species Distribution
Source: PLoS One. 2013 Jan 11;8(1):e53646. doi: 10.1371/journal.pone.0053646 (PMC3548625; doi:10.1371/journal.pone.0053646)

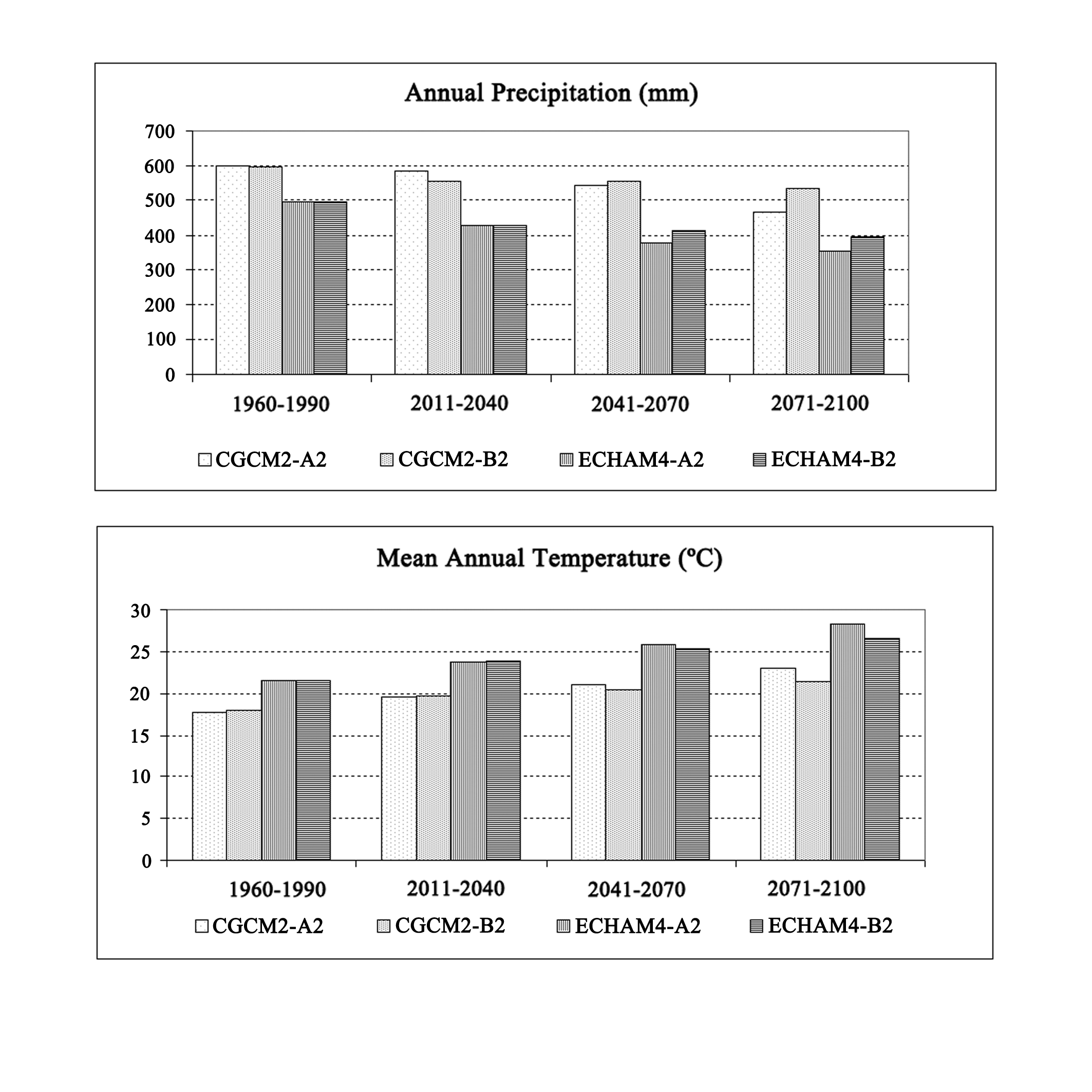

Supplement: Figure S1 — Precipitations and temperatures. Annual precipitations and mean annual maximum temperatures for each period and combination of circulation model and scenario used. (TIF) [file pone.0053646.s001.tif]
