# Supplementary material for: Estimating How Inflated or Obscured Effects of Climate Affect Forecasted Species Distribution
Source: PLoS One. 2013 Jan 11;8(1):e53646. doi: 10.1371/journal.pone.0053646 (PMC3548625; doi:10.1371/journal.pone.0053646)
Supplement: Table S1 — Factors and variables. Explanatory factors and variables used to model the species distributions of Márquez et al 2011 [19]. (DOC) [file pone.0053646.s002.doc]

| **Factors** | **Code** | **Variables** |
| --- | --- | --- |
| ***Spatial situation*** | ***La*** | Latitude (ºN) ^(1)^ |
|  | ***Lo*** | Longitude (ºE) ^(1)^ |
| ***Topography*** | ***A*** | Mean altitude (m) ^(2)^ |
|  | ***S*** | Slope (º) (calculated from altitude) |
|  | ***SE*** | Southward exposure degree^(3)^ |
|  | ***WE*** | Westward exposure degree^(3)^ |
| ***Human activity*** | ***DHi*** | Distance to the nearest highway (km) ^(1)^ |
|  | ***U100*** | Distance to the nearest urban centre with more than 100 000 inhabitants (km) ^(1)^ |
|  | ***U500*** | Distance to the nearest urban centre with more than 500 000 inhabitants (km) ^(1)^ |
|  | ***HPd*** | Human population density in 2000 (number of inhabitants/Km^2^)^(4)^ |
| ***Climatic*** |  | |
|  | ***PAn*** | Annual precipitation (mm)^(5)^ |
|  | ***PSp*** | Spring precipitation (mm)^(5)^ |
|  | ***PSu*** | Summer precipitation (mm)^(5)^ |
|  | ***PAu*** | Autumn precipitation (mm)^(5)^ |
|  | ***PWi*** | Winter precipitation (mm)^(5)^ |
|  | ***TAn*** | Annual maximum temperature^(5)^ |
|  | ***TJa*** | January maximum temperature^(5)^ |
|  | ***TJu*** | July maximum temperature^(5)^ |
|  | ***TSp*** | Spring maximum temperature^(5)^ |
|  | ***TSu*** | Summer maximum temperature^(5)^ |
|  | ***TAu*** | Autumn maximum temperature^(5)^ |
|  | ***TWi*** | Winter maximum ^(5)^ |

Sources: ^(1)^IGN (1999); ^(2)^US Geological Survey (1996); ^(3)^Shuttle Radar Topography Mission (SRTM), Farr and Kobrick, 2000; ^(4)^ORNL (2001) LandScan 2000 Global Population Database. Oak Ridge National Laboratory (ORNL), Oak Ridge, Tennessee; ^(5)^Agencia Estatal de Meteorología of Spain (AEMET), Ministerio de Medio Ambiente (http://www.aemet.es/es/elclima/cambio_climat/escenarios).

**References**

Agencia Estatal de Meteorología of Spain (AEMET) Ministerio de Medio Ambiente (http://www.aemet.es/es/elclima/cambio_climat/escenarios)

I.G.N. 1999. Mapa de carreteras. Penı´nsula Ibe´rica, Baleares y Canarias. Inst. Geogra´fico Nacional/Ministerio de Fomento, Madrid.

Farr, T. G. and Kobrick, M. 2000. Shuttle Radar Topography Mission produces a wealth of data. EOS Trans. Am. Geophys. Union 81: 583 585.

ORNL 2001. LandScan 2000 Global Population Database. Oak Ridge National aboratory (ORNL), Oak Ridge, TN.

US Geological Survey 1996. GTOPO30. Land Processes Distributed Active Archive Center (LP DAAC), EROS Data Center. <http://edcdaac.usgs.gov/gtopo30/gtopo30.asp>>
